# Rickettsiae Induce Microvascular Hyperpermeability via Phosphorylation of VE-Cadherins: Evidence from Atomic Force Microscopy and Biochemical Studies

**DOI:** 10.1371/journal.pntd.0001699

**Published:** 2012-06-12

**Authors:** Bin Gong, Liang Ma, Yan Liu, Qinyu Gong, Thomas Shelite, Donald Bouyer, Paul J. Boor, Yong Sun Lee, Andres Oberhauser

**Affiliations:** 1 Department of Pathology, University of Texas Medical Branch at Galveston, Galveston, Texas, United States of America; 2 Center for Biodefense and Emerging Infectious Diseases, University of Texas Medical Branch at Galveston, Galveston, Texas, United States of America; 3 Department of Neurosciences and Cell Biology, University of Texas Medical Branch at Galveston, Galveston, Texas, United States of America; 4 Department of Biochemistry and Molecular Biology, University of Texas Medical Branch at Galveston, Galveston, Texas, United States of America; 5 Sealy Center for Structural Biology and Molecular Biophysics, University of Texas Medical Branch at Galveston, Galveston, Texas, United States of America; University of Texas Medical Branch, United States of America

## Abstract

The most prominent pathophysiological effect of spotted fever group (SFG) rickettsial infection of microvascular endothelial cells (ECs) is an enhanced vascular permeability, promoting vasogenic cerebral edema and non-cardiogenic pulmonary edema, which are responsible for most of the morbidity and mortality in severe cases. To date, the cellular and molecular mechanisms by which SFG *Rickettsia* increase EC permeability are largely unknown. In the present study we used atomic force microscopy (AFM) to study the interactive forces between vascular endothelial (VE)-cadherin and human cerebral microvascular EC infected with *R. montanensis*, which is genetically similar to *R. rickettsii* and *R. conorii*, and displays a similar ability to invade cells, but is non-pathogenic and can be experimentally manipulated under Biosafety Level 2 (BSL2) conditions. We found that infected ECs show a significant decrease in VE-cadherin-EC interactions. In addition, we applied immunofluorescent staining, immunoprecipitation phosphorylation assay, and an *in vitro* endothelial permeability assay to study the biochemical mechanisms that may participate in the enhanced vascular permeability as an underlying pathologic alteration of SFG rickettsial infection. A major finding is that infection of *R. montanensis* significantly activated tyrosine phosphorylation of VE-cadherin beginning at 48 hr and reaching a peak at 72 hr p.i. *In vitro* permeability assay showed an enhanced microvascular permeability at 72 hr p.i. On the other hand, AFM experiments showed a dramatic reduction in VE-cadherin-EC interactive forces at 48 hr p.i. We conclude that upon infection by SFG rickettsiae, phosphorylation of VE-cadherin directly attenuates homophilic protein–protein interactions at the endothelial adherens junctions, and may lead to endothelial paracellular barrier dysfunction causing microvascular hyperpermeability. These new approaches should prove useful in characterizing the antigenically related SFG rickettsiae *R. conorii* and *R. rickettsii* in a BSL3 environment. Future studies may lead to the development of new therapeutic strategies to inhibit the VE-cadherin-associated microvascular hyperpermeability in SFG rickettsioses.

## Author Summary

Rickettsial diseases are serious human infections. Some spotted fever group (SFG) rickettsial pathogens are bioterror agents. A major clinical hallmark of SFG rickettsial disease is the infection of endothelial cells leading to enhanced vascular permeability. Previous studies show that SFG rickettsiae cause dose-dependent hyperpermeability, which was associated with disruption of intercellular adherens junctions (AJs). The underlying molecular mechanism by which the junctional complexes are disrupted, ultimately causing changes in the endothelial paracellular milieu during rickettsial infection, remains largely unclear. The available evidence suggests that inflammatory stimuli can trigger tyrosine phosphorylation of various components of AJs, mainly the vascular endothelial–cadherin (VE-cadherin). This causes gaps at AJs, partially due to phosphorylation-induced destabilization of VE-cadherins at the plasma membrane and increased endocytosis, greatly increasing paracellular leaks. Here, we hypothesize that infection by SFG rickettsiae induces endothelial cells to develop altered VE-cadherin in association with phosphorylation of tyrosine residues. Utilizing nano-mechanical studies with atomic force microscopy and biochemical analysis of the major AJ protein VE-cadherin, we have implicated that phosphorylation of VE-cadherin directly attenuates homophilic interactions between VE-cadherins. The experimental approach advances a new way of studying rickettsial infection. This strategy should prove useful in uncovering novel therapeutic strategies for virulent arthropod-borne rickettsioses.

## Introduction

Spotted fever group (SFG) rickettsioses are composed of over 25 species of rickettsiae that are causative agents of a wide spectrum of diseases, ranging from the virulent Rocky Mountain spotted fever (*Rickettsia rickettsii*) and severe systemic Mediterranean spotted fever (*R. conorii*) to the recently identified *R. parkeri* rickettsiosis (*R. parkeri*) and non-pathogenic *R. montanensis* [1], [2]. The main target cells of SFG rickettsiae are the endothelial cells that line the entire vasculature [3]–[5]. The most prominent pathophysiological effects of rickettsial infection are increased microvascular permeability, promoting vasogenic cerebral edema and non-cardiogenic pulmonary edema, which are responsible for most of the severity and mortality in Rocky Mountain spotted fever and Mediterranean spotted fever [6]. The cellular and molecular mechanisms by which *Rickettsia* increase endothelial cell permeability are largely unknown. Previous studies show that *R. rickettsii* and *R. conorii* cause dose-dependent hyperpermeability, which was associated with disruption of intercellular adherens junctions (AJs) after infection [5], [7], [8]. The underlying molecular mechanism by which the junctional complexes are disrupted, ultimately causing changes in the endothelial paracellular milieu during rickettsial infection, remains unclear [6], [9].

The available evidence suggests that inflammatory stimuli such as histamine, tumor necrosis factor (TNF), and vascular endothelial growth factor (VEGF) can trigger tyrosine phosphorylation of various components of AJs, mainly the vascular endothelial–cadherin (VE-cadherin), β-catenin, and p120-catenin complex, consequently dissociating catenins from the complex [10]–[12]. This causes gaps at AJs, partially due to phosphorylation-induced destabilization of VE-cadherins at the plasma membrane and increased endocytosis [10], [13], [14], greatly increasing paracellular leaks in cultured endothelial cells [15].

Here, we hypothesize that infection by SFG rickettsiae induces endothelial cells to develop altered junctional protein VE-cadherin in association with phosphorylation of tyrosine residues, so that the Ca^2+^-dependent, homophilic *cis* and *trans* interactions with their extracellular regions [16], [17] are affected or even eliminated, resulting in aberrant properties of junctional complexes. In order to test this hypothesis, detailed information about the biomechanical properties of protein–protein interactions as well as protein–cell interactions at the molecular level is required. Atomic force microscopy (AFM) is ideally suited for these studies because it has a unique capability to measure the interactive forces between receptors and ligands with piconewton resolution [18]–[22]. Within the last decade, this technique has been developed to exert and measure inter- or intra-molecular forces, revealing detailed insights into the functional mechanics of biomolecules [23]–[27]. AFM has been employed to study different cadherin interactions *in vitro*, in order to mimic different aqueous physiological conditions *in vivo* [27]–[30].

In the present study, we used single-molecule AFM techniques to study the nanomechanical properties of the interactive forces between VE-cadherin and living human cerebral microvascular endothelial cells upon infection with *R. montanensis*, which is genetically similar to *R. rickettsii* and *R. conorii* and displays a similar ability to invade cells *in vitro* and can be experimentally manipulated in the Biosafety Level 2 (BSL2) environment [1], [2], [31]. In addition to AFM techniques, we applied routine immunofluorescent (IF) staining, immunoprecipitation (IP) phosphorylation assay, and *in vitro* endothelial permeability assay to study the biochemical mechanisms that may participate in the enhanced vascular permeability as an underlying pathologic alteration of SFG rickettsial infection. Our experiments help elucidate the molecular mechanism by which SFG rickettsial infection may trigger tyrosine phosphorylation of VE-cadherins, thus destabilizing homophilic molecular interactions at AJs and altering endothelial biophysical features to enhance paracellular leaks.

## Materials and Methods

### Reagents

Recombinant human VE-cadherin Fc chimera was purchased from R&D Systems (Minneapolis, MN). Cell culture medium Prigrow I and fetal bovine serum were obtained from Applied Biological Materials (Richmond, BC, Canada). Unless otherwise indicated, all reagents were purchased from Thermal Fisher Scientific (Waltham, MA).

### *Rickettsia* purification

To allow us to employ BSL2 procedures, we utilized a BSL2 rickettsial species, *R. montanensis* (strain M/5–6), was used for the present study, obtained from the laboratory of David H. Walker. A 10% yolk sac suspension of *R. montanensis* from infected eggs diluted in sucrose-phosphate-glutamate (SPG) buffer (0.218 M sucrose, 3.8 mM KH_2_PO_4_, 7.2 mM K_2_HPO_4_, 4.9 mM monosodium l-glutamic acid, pH 7.0) was propagated through two passages in Vero cells [32], [33]. *R. montanensis* cells were harvested from 180-cm^2^ tissue culture flasks containing confluent monolayers of infected Vero cells. The infected Vero cells were harvested from each flask surface with scraper, diluted in 10 ml of supplemented medium, and centrifuged at approximately 13,000×*g* for 5 min at room temperature. The pellet from each flask was suspended in 15 ml of supplemented media, and was transferred to a precooled 50-ml tube containing 5 g of 3-mm glass beads, and vortexed vigorously for 30 s in order to disrupt the Vero cells. Vortexing was repeated two times with 60-s intervals of incubation on ice between each 30-s vortexing. The lysates were centrifuged at approximately 800× *g* for 10 min to remove unbroken Vero cells and cellular debris. The supernatant, containing released *R. montanensis* cells, was transferred to a tube, and the rickettsiae were pelleted by centrifugation at 15,000×*g* for 25 min at 4°C. Purified rickettsiae were frozen in SPG buffer at −80°C. Rickettial content of the frozen stocks was determined by plaque assay and TCID_50_ assays on Vero cells, and yielded approximately 1×10^9^ bacterial cells per ml. Uninfected Vero cells were processed by the same procedure as normal control material.

### Cell culture

Immortalized human cerebral microvascular endothelial cells (h-CMEC; Applied Biological Materials, Richmond, BC, Canada) were grown in Prigrow I medium supplemented with 10% heat-inactivated fetal bovine serum in 5% CO_2_ at 37°C. All experiments were performed between passages 15 and 18, and cells were fed with Prigrow I medium with 1% fetal bovine serum.

h-CMEC were cultured on round glass coverslips (12 mm diameter, Ted Pella, Redding, CA) for AFM studies and IF assay until confluent at 90%. The cells were then infected with *R. montanensis* at a multiplicity of infection (MOI) of 10. After 24, 48, and 72 h, the cells on the coverslips were washed three times in phosphate-buffered saline (PBS) before the downstream studies were performed.

### Atomic force microscopy

The mechanical properties between VE-cadherin functionalized AFM tips and cell monolayers were studied using AFM that consisted of a detector head (Digital Instruments, Tonawanda, NY) mounted on top of a single axis piezoelectric positioner with a strain gauge sensor (P841.10, Physik Instrumente, Auburn, MA). This system has a z-axis resolution of a few nm and can measure forces in the range of 5–10,000 pN [34]. The monitoring of the force reported by the cantilever and the control of the movement of the piezoelectric positioners are achieved by means of two data acquisition boards (PCI 6052E, PCI 6703, National Instruments) and controlled by custom-written software (Wavemetrics, Portland, OR). In order to measure the interactive forces, we used cantilevers with a 10 µm latex bead glued to the tip (Novascan Technologies, Ames, IA). We incubated the cantilevers with 50 µl of recombinant human VE-cadherin/Fc (R&D Systems, Minneapolis, MN) at 100 µg/mL in 0.1 M NaHCO_3_ (pH 8.6) overnight at 4°C. Unbound proteins were removed by rinsing with PBS. Bovine serum albumin (BSA, Sigma, St. Louis, MO) at 500 µg/ml in PBS was used to block the exposed surface of the latex bead. The spring constant of each individual cantilever was calculated using the equipartition theorem [21]. The cantilever spring constant varied between 20–50 pN/nm. Interactive forces were measured by pressing the cantilever onto the cell monolayer for ∼500 ms and then stretching for several hundred nm. We used a serum-free Hank's Balanced Salt Solution (HBSS) supplemented with 10 mM HEPES, 2 mM CaCl_2_ and 1 mM glucose. In the experiments using blocking antibodies, cells were pretreated with different antibodies (25 µg/ml) for 15 min before the AFM measurements. Unless noted, the pulling speed of the different force-extension curves was about 1.0 µm/s.

### Endothelial cell permeability assay

The permeability of h-CMECs upon infection with *R. montanensis* at a MOI of 10 was determined using an *in vitro* vascular permeability assay (Millipore, Billerica, MA) as previous described [12], [14], [35], [36]. Briefly, h-CMECs were seeded onto type I rat-tail collagen-coated polycarbonate Transwell filters (6.5-mm diameter and 3-µm pore size; Millipore, Billerica, MA) and confluent monolayers were inoculated with *R. montanensis* or mock-infected control material cells. At different time points post-infection (p.i.), hCMEC permeability was assessed by adding 0.5 mg/ml of fluorescein isothiocyanate (FITC)-dextran (40 kDa; Sigma, St. Louis, MO) to the top chamber above the filter. After 3 hours, FITC-dextran present in the bottom compartment was assayed by using a BioTek Synergy 2 multi-mode microplate reader (485 nm excitation, 530 nm emission). The fold-change in fluorescence intensity over the basal permeability of monolayers was used as an indicator of paracellular permeability of assessed monolayers. Experiments were performed in sets of four.

### Immunofluorescence (IF)

Cells were fixed with cold methanol at 24, 48, or 72 h after infection. Each experiment was repeated three times. The primary antibodies, a mouse monoclonal IgG against VE-cadherin (1/500) (Clone TEA1/31, Meridian Life Science, Saco, ME) and a rabbit polyclonal IgG antibody against SFG rickettsiae (1∶5000), were added and incubated for 2 h. VE-cadherins and rickettsiae were detected with secondary goat anti-mouse Alexa 488 and goat anti-rabbit Alexa 594 conjugated antibodies (Invitrogen, Carlsbad, CA), respectively. IF images were taken and analyzed with an Olympus BX51 imaging system.

### Western immunoblots

In experiments following IF studies, *in vitro* cellular expression of VE-cadherin was analyzed by Western immunoblotting according to established methods [37], [38]. After infection with *R. montanensis* for 24, 48, or 72 hr in T75 flasks, whole-cell extracts of infected and mock control cells were prepared by lysis in RIPA buffer (Santa Cruz Biotechnology, Santa Cruz, CA) containing 1× PBS, 1% Nonidet P-40, 0.5% sodium deoxycholate, 0.1% sodium dodecyl sulfate (SDS), aprotinin, and phenylmethylsulfonyl fluoride (PMSF). The concentration of total protein was determined before equal amounts of soluble protein (50 ug/lane) were subjected to SDS–polyacrylamide gel electrophoresis (SDS-PAGE) (10% acrylamide) (Invitrogen, Carlsbad, CA). Proteins were transferred onto a polyvinylidene difluoride (PVDF) membrane and then incubated with mouse monoclonal anti-VE-cadherin antibody (dilution 1∶1000; Meridian Life Science, Saco, ME), followed by incubation with a secondary antibody at 1∶2000 for 30 min. Blots were visualized by using a chemiluminescence kit (Pierce, Rockford, IL). Data were analyzed densitometrically using 1D scan EX software (BD Biosciences, Rockville, MD). A Western blot for α-tubulin served as loading control to verify equal loading and transfer.

### Immunoprecipitation (IP) phosphorylation assay [39]

To study VE-cadherin phosphorylation, cell lysates were prepared with an ice-cold RIPA lysis buffer (the same as for Western immunoblot assays). After centrifugation at 12,000×*g* for 20 min, the protein supernatant was collected. Equal amounts of protein with optimal Dynabead Protein G (Invitrogen, Carlsbad, CA) conjugated with anti-VE-cadherin antibody were incubated for 2 h at room temperature. The Dynabead-antibody-antigen complex pellets were precipitated and separated using DynaMag-2 (Invitrogen, Carlsbad, CA). The pellet was washed three times with PBS, and resuspended in 20 µL of SDS sample buffer (Invitrogen, Carlsbad, CA) and heated for 10 min at 70°C. Samples were then separated by gel electrophoresis followed by immunoblotting. A mouse monoclonal anti-phosphotyrosine antibody, 4G10 (Millipore, Billerica, MA) was used at dilution of 1∶500 for detection of proteins containing phosphotyrosine. All experiments were performed in sets of three.

### Statistical analysis

Values are reported as mean ± SD. The data were analyzed using Student's paired *t*-test (Sigmaplot, Sigma Stat, Jandel Scientific Software, San Rafael, CA). Statistical significance was determined at *P*<0.05.

## Results

### SFG rickettsial infection affects endothelial AJ integrity and enhances paracellular permeability

**Figure 1:**
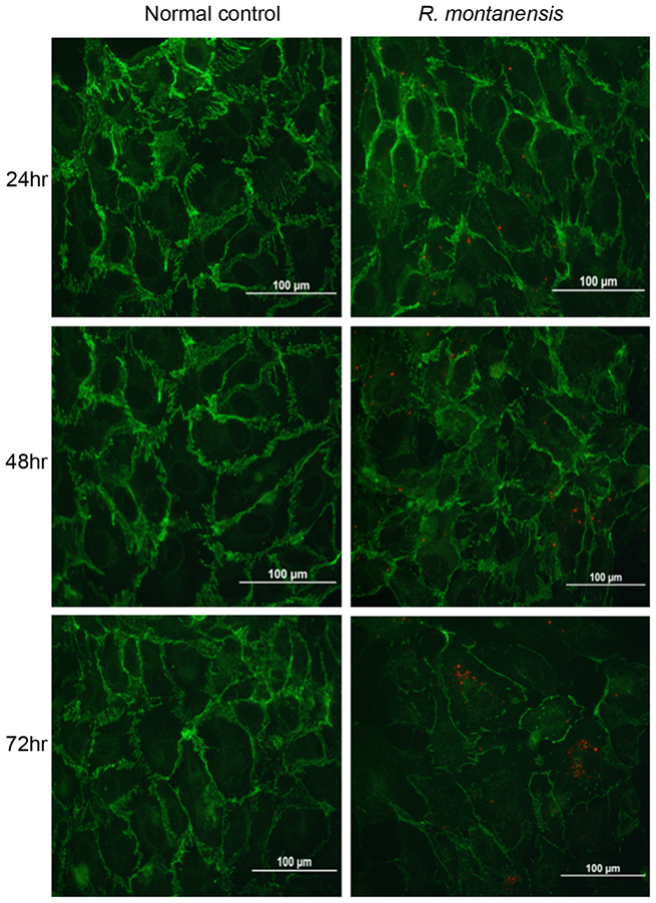
Immunofluorescence studies show rickettsiae (red) located in human cerebral microvascular endothelial cells at 24, 48 and 72 hr after infection. Dual immunofluorescence staining of SFG rickettsiae (red) and VE-cadherin (green) using dual wave lengths filter system reveals that, compared to normal controls, *R. montanensis* infection (10 MOI) resulted in degradation of the density of VE-cadherin, suggesting disruption in the continuity of VE-cadherin at neighbouring areas at 72 hr post-infection.

The abnormal VE-cadherin expression induced upon infection of rickettisae was visualized under fluorescence microscopy. At earlier time points, no detectable difference was noted in IF studies of VE-cadherin compared to normal controls, although rickettsiae were detected at 24 hr and 48 hr post-infection (Figure 1). As the infection progressed, VE-cadherin's distribution appeared disrupted after 72 hr at endothelial cell contacts in confluent cell layers when compared to controls (Figure 1).

**Figure 2:**
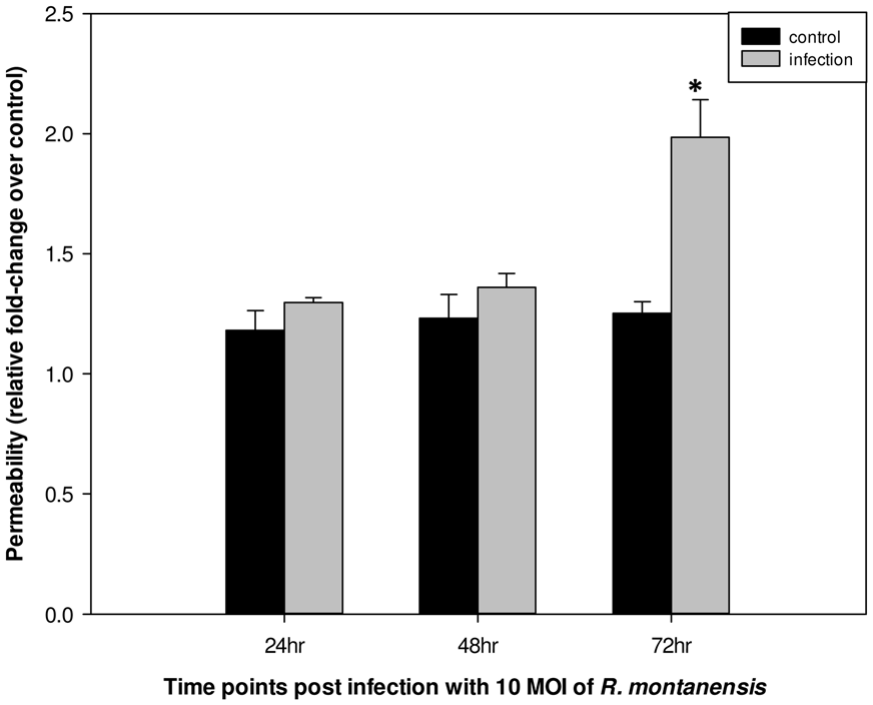
*R. montanensis* infection enhanced human cerebral microvascular endothelial cell permeability. Endothelial cells were seeded on type I rat-tail collagen-coated polycarbonate transwell filters and infected with *R. montanensis* at an MOI of 10 in triplicate, or mock infected. After 24, 48, and 72 hr, FITC-dextran was added to the upper chamber medium, and the presence of FITC dextran in the lower chamber was quantified after 3 hr. The results are expressed as the fold-increase in monolayer permeability over basal permeability levels (* p<0.05).

To determine if disorganized or reduced VE-cadherin at endothelial AJs are relevant to endothelial paracellular barrier dysfunction, we assessed cell permeability by an *in vitro* vascular endothelial cell permeability assay. As seen in Figure 2, in h-CMEC monolayers, infection of *R. montanensis* induced a 1.58-fold increase in para-endothelial cell permeability at 72 hr post-infection compared to control. There was no significant change in hCMEC monolayers at 24 hr and 48 hr post-infection compared to normal controls.

### SFG rickettsial infection causes tyrosine phosphorylation of VE-cadherin, leading to increased instability of VE-cadherin at inter-endothelial interactions

**Figure 3:**
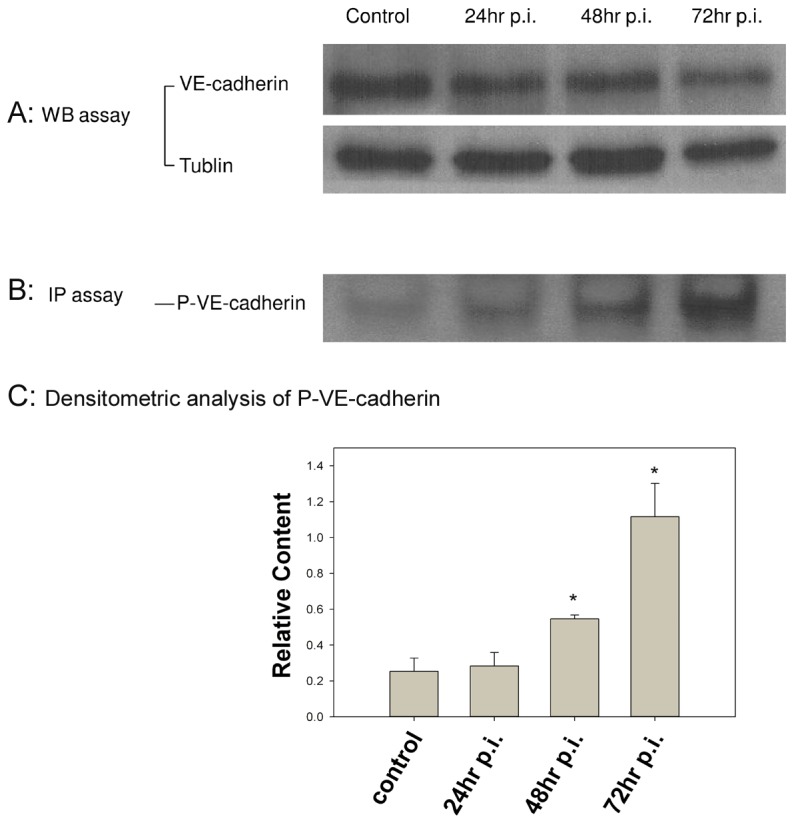
*R. montanensis* enhanced VE-cadherin tyrosine phosphorylation. Human cerebral microvascular endothelial cells were mock-infected (control) or infected with *R. montanensis* at a MOI of 10. At 24, 48, or 72 hr post-infection, cells were harvested for Western immunoblot or immunoprecipitated with anti-VE-cadherin antibody using a magnetic bead system. **3A**). The Western immunoblot for α-tubulin served as a control to verify equal loading and transfer. There was no significant difference in VE-cadherin expression detected by Western immunoblot. **3B and 3C**). Loading of VE-cadherin was detected by anti-VE-cadherin antibody. The normalized relative densities from IP phosphorylation assay showed a 2.16- and 4.48-fold increase in phosphorylation of VE-cadherin (P-VE-cadherin) at 48 hr and 72 hr post-infection, respectively, compared to control cells (* p<0.05). These data are representative of three independent experiments.

To determine the possible biochemical basis of increased endothelial permeability, we examined VE-cadherin using Western immunoblotting. Initial studies failed to reveal any alterations in expression of general VE-cadherin between control and infected experimental monolayers at 24, 48, and 72 h post-infection (Figure 3A). Therefore, we used an IP-phosphorylation assay to focus on tyrosine phosphorylation of VE-cadherin because phosphorylation is thought to be an important event leading to destabilization of the AJ complex [10], [11], [14]. Infection with *R. montanensis* at a MOI of 10 stimulated increased tyrosine phosphorylation of VE-cadherin at 48 hr, with even greater phospholyration at 72 hr post-infection (Figure 3B and 3C). This time corresponds to the increased endothelial permeability, suggesting that modulating VE-cadherin activity through phophorylation is one of the mechanisms regulating VE-cadherin-related endothelial monolayer paracellular permeability.

### AFM measurements of the adhesion forces between VE-cadherin and endothelial cells

For AFM experiments, human VE-cadherins were immobilized on beads attached to cantilever tips. Subsequently, this functionalized cantilever was gently brought into contact with the surface of a confluent monolayer of human cerebral microvascular ECs. The maximum compression force was set to approximately 200 pN. The contact time was critical in this study, and was constantly kept about 0.5 s before the cantilever was retracted at a constant pulling speed of 0.5 um/s. AFM mimicked the binding of intramembrane VE-cadherins between the ECs under physiological conditions. By monitoring the cantilever deflection and retraction cycle, the binding, stretching, and rupture of VE-cadherin-VE-cadherin complexes can be monitored in terms of forces and distance as a function of time.

**Figure 4:**
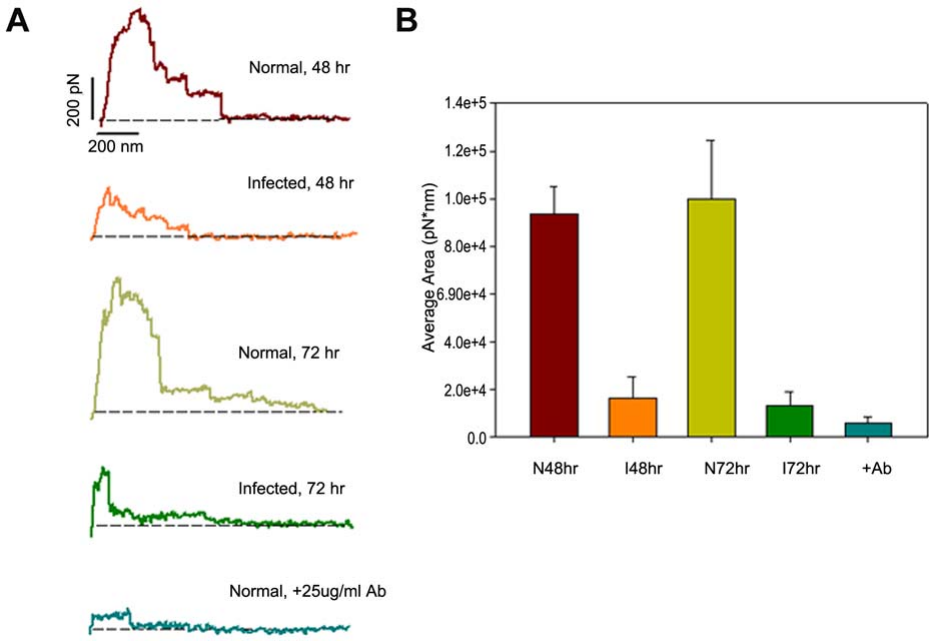
AFM measurements of the de-adhesion forces between VE-cadherin and endothelial cells. **4A**). Typical force-extension curves obtained between cells and VE-cadherin coated AFM tips. The dashed lines indicate zero force. The experiments were carried out in uninfected and infected cells at different time points post-*R. montanensis* infection (48 hr and 72 hr). **4B**). Work of de-adhesion between VE-cadherin beads and endothelial cells at different time points post-infection. *R. montanensis*-infected cells required a significantly lower level of average work to break the interaction compared with uninfected cells. The addition of a monoclonal antibody against VE-cadherin significantly blocked the VE-cadherin-endothelial cell interaction. Ab: anti-VE-cadherin antibody.

Typical force-extension patterns of the interactions between VE-cadherin and ECs are shown in Figure 4A. The force curves represent the force changes on the cantilever as a function of its travel distance. By integrating the areas underneath the force curve and above the baseline (dashed lines to present zero force, Figure 4A), we calculated the work that is required to break any interactive bonds between the cantilever and the ECs. Normal ECs always show a strong binding to the VE-cadherin functionalized cantilever. To our surprise, rickettsial-infected ECs showed a dramatic decrease in binding affinity to VE-cadherin as early as 48 hrs post-infection. The average work from infected cells decreased to ∼20% of that of uninfected cells (Figure 4B). The level of work in infected cells remained low after 72 hrs.

To confirm the specificity of this VE-cadherin-EC interaction, several control experiments were performed. For example, an excess of antibodies to VE-cadherin was added, or BSA was added to block the interactions between the VE-cadherin and ECs. As shown in Figure 4, the presence of a blocking monoclonal antibody against VE-cadherin (25 µg/ml) resulted in a significant decrease in the adhesion interaction between normal cells and the probing cantilever. The detected force was small, only about 30 pN, which is close to the detection limit of our instrument (∼20 pN). Another control experiment was to probe the normal cells with a cantilever covered by BSA at 100 µg/ml in PBS. No force was detected in this experiment since the exposed surface of the cantilever was completely blocked by BSA (data not shown).

## Discussion

SFG rickettsial diseases are serious human infections. Some SFG rickettsial pathogens are bioterror agents [40]. A major clinical hallmark of SFG rickettsial disease is the infection of EC leading to enhanced vascular permeability [6]. The cellular and molecular mechanisms by which SFG rickettsiae increase endothelial permeability are largely unknown [4]. The endothelial cells that line all blood vessels function to regulate the influx and efflux of solutes and fluids between the vessel lumen and the surrounding interstitium. The movement of vessel contents is mediated by two broad mechanisms, the paracellular and transcellular routes. Relatively little is known about the role of the second route in microvascular hyperpermeability during inflammation [14]. The paracellular pathway, which is generally accepted to be dominant in inflammatory pathological states, is controlled by the dynamic opening and closing of endothelial junctions, mainly mediated by transmembrane proteins VE-cadherin at AJs and claudin at tight junctions (TJs) [10], [41], [42]. VE-cadherin initiates cell-cell adhesion and promotes its maintenance through its transmembrane domains [17]. VE-cadherin may also form a signaling complex through its cytoplasmic tail, interacting with β-catenin and p120-catenin [43]. However, it is hard to clearly separate these two aspects. VE-cadherins are linked to a large variety of intracellular partners that mediate intracellular signaling and modulate the organization of the actin cytoskeleton to provide the dynamic forces necessary for appropriate tissue morphogenesis [10]. VE-cadherin-deficient mice die at mid-gestation due to defective vascular remodeling [44]. The primitive vascular plexus initially forms, but beyond embryonic day 9 these vessels regress and disintegrate. VE-cadherin-blocking antibodies disrupt cell-cell adhesion, increase permeability, and enhance transmigration of leukocytes [45], [46]. However, VE-cadherin's role in the mechanism responsible for enhanced microvascular permeability during SFG rickettsioses needs to be elucidated.

In an earlier study, a remarkable observation was made regarding discontinuities in the endothelial localization of AJ proteins after a prolonged period of *R. conorii* infection [9]. Similar findings were made by our group using IF studies in mouse models of intravenous infection by *R. conorii*. Endothelial cells lining cerebral and pulmonary microcirculation display significantly diminished AJ and TJ proteins at day 5 after infection with a lethal dose of rickettsiae (*unpublished observations*). Furthermore, in an *in vitro* functional study, enhanced microvascular endothelial permeability has been described, which is correlated with dissociation of AJs (β-catenin and p120) during 24, 48, and 72 hr post-infection by *R. rickettsii* [7]. In the present study using a human cerebral mirovascular endothelial model, we observed aberrant structures of inter-ECs VE-cadeherin at 72 hr post-infection by *R. montanensis*, in which enhanced microvascular permeability was documented using an *in vitro* endothelial cell permeability assay.

VE-cadherins engage in Ca^2+^-dependent homophilic interactions in which a VE-cadherin molecule on one cell binds to an identical VE-cadherin molecule on an adjacent cell [43]. After binding, cadherins aggregate laterally in *trans* and *cis* at cell–cell junctions and form a zipper-like structure along the cell border that promotes tight adhesion between endothelial cells [16], [17]. In the present study, we used AFM to directly examine alterations in protein-protein adhesion forces that underlie this paracellular dysfunction following SFG rickettsial infection. AFM experiements revealed a dramatic reduction in the interactive forces between VE-cadherin and EC after 48 hr of infection. This decreased protein-EC interaction took place prior to the enhanced microvascular permeability detected by *in vitro* endothelial permeability assay at 72 hr p.i. This fact indirectly supports the idea that Ca^2+^-dependent homophilic interactions between VE-cadherin molecules on adjacent cells are the target during SFG rickettsial infection-induced endothelial hyperpermeability.

There are many mechanisms that regulate VE-cadherin, including modulating VE-cadherin activity through phosphorylation and controlling VE-cadherin availability at the endothelial surface [10], [47]. Stimuli such as histamine, thrombin, tumor necrosis factor (TNF), and vascular growth factor (VEGF) induce tyrosine phosphorylation of VE-cadherin, in which Src and Rac play a role as key pathway mediators to promote kinase-regulated phosphorylation of VE-cadherin on different residues attenuating stability at endothelial AJs [10], [48]. Evidence has been established that mediators of inflammation signal through Src and Rac to trigger the tyrosine phosphorylation of VE-cadherin, leading to the endocytosis of VE-cadherin in a β-arrestin-dependent fashion [49]. Thus, kinase-mediated phosphorylation coordinates with the destablizing barrier function of VE-cadherin at endothelial AJs. By competing with phosphorylate kinase, binding of p120-catenin may prevent VE-cadherin endocytosis from the plasma membrane, stabilizing it at the endothelial AJ [50]. Previous studies have demonstrated that the catenin class of proteins, β-catenin and p120-catenin, dissociate from the interendothelial cell junctions in response to SFG rickettsial infections [7], [9]. Furthermore, a study using *in vitro* human endothelial-targeted *R. rickettsii* and human cerebral microvascular endothelial cells showed that the addition of pro-inflammatory stimuli essential to rickettsial immunity enhances rickettsia-induced microvascular permeability in a dose-dependent manner [7]. Taken together, this evidence suggests that SFG rickettsial infection may cause endothelial paracellular barrier dysfunction in association with phosphorylation of VE-cadherin, thus destabilizing endothelial AJs. A major finding of the present study is that upon infection by SFG rickettsiae, tyrosine phosphorylation of VE-cadherin was activated in human cerebral microvascular endothelial cells, which started at 48 hr and increased at 72 hr post-infection, although no difference was detected for general VE-cadherin expression at the same time. Given that the *in vitro* endothelial permeability assay showed enhanced microvascular permeability at 72 hr post-infection and the AFM studies showed a dramatic reduction in the adhesive force between VE-cadherin and endothelial cells at 48 hr, we suggest that upon infection by SFG rickettsiae, phosphorylation of VE-cadherin directly attenuates homophilic protein-protein interactions at the endothelial AJs, leading to endothelial paracellular barrier dysfunction and microvascular hyperpermeability.

In the present study, we present data to support the association between phosphorylation of endothelial AJ proteins and enhanced microvascular permeability during SFG rickettsial infection. However, it is not established whether activated phosphorylation is a direct consequence of rickettsial infection of the endothelial microvasculature, or whether it is a consequence of less specific physiological responses such as inflammation. We will utilize atomic force microscopy in future studies involving pathogenic SFG *R. conorii* and *R. rickettsii* to help to address the potential role of rickettsiae as a trigger mechanism to alter major AJ components that affect vascular permeability.

In summary, our results indicate that phosphorylation of VE-cadherin directly attenuates homophilic interactions between VE-cadherins. Our nano-mechanical and biochemical studies of the major endothelial AJ protein VE-cadherin have implicated attenuated VE-cadherin-endothelial cell interaction as an underlying cause of enhanced microvascular permeability that occurs at one prolonged stage upon infection by *R. montanensis*. Our experimental approach advances a new way of studying rickettsial infection and will allow similar studies of the closely related SFG rickettsiae *R. conorii* and *R. rickettsii*. This strategy should prove useful in uncovering novel therapeutic strategies for virulent arthropod-borne rickettsioses.
